# Adherence with brand versus generic bisphosphonates among osteoporosis patients: a new-user cohort study in the French National Healthcare Insurance database

**DOI:** 10.1038/s41598-020-64214-x

**Published:** 2020-05-04

**Authors:** Marie Viprey, Yufeng Xue, Aurélie Rousseau, Cécile Payet, Roland Chapurlat, Pascal Caillet, Alexandra Dima, Anne-Marie Schott

**Affiliations:** 1 Hospices Civils de Lyon, Pôle de Sante Publique, Lyon, France; 2 Univ. Lyon, Université Claude Bernard Lyon 1, HESPER EA 7425, Lyon, France; 3 Centre Hospitalier de Bourg en Bresse, Service pharmaceutique, Bourg en Bresse, France; 4 Université de Lyon, INSERM UMR 1033, Lyon, France; 5 Hospices Civils de Lyon, Hôpital Edouard Herriot, Service de Rhumatologie, Lyon, France; 6 CHU de Nantes, Service de Pharmacologie Clinique, Nantes, France

**Keywords:** Bisphosphonates, Rheumatology

## Abstract

Several studies documented declines in treatment adherence with generic forms of oral bisphosphonates in osteoporosis compared to branded forms, while others did not support this relation. Our aim was to compare medication adherence with brand versus generic forms of oral bisphosphonates. A new-user cohort study was conducted using routinely collected administrative and healthcare data linked at the individual level extracted from a nationwide representative sample of the French National Healthcare Insurance database. We included all patients aged 50 and older, new users of oral bisphosphonates for primary osteoporosis between 01/01/2009 and 31/12/2015. Two components of adherence were measured: implementation (continuous multiple-interval measure of medication availability version 7; CMA7) and persistence (time to discontinuation). The sample was composed of 1,834 in the “brand bisphosphonate” group and 1,495 patients in the “generic bisphosphonate” group. Initiating oral bisphosphonate treatment with brand was associated with a higher risk of discontinuation within 12 months (Hazard Ratio = 1.08; 95%CI = [1.02;1.14]). The risk of good implementation (CMA7 ≥ 0.90) was significantly lower in “brand bisphosphonate” group (Risk Ratio = 0.90; 95%CI = [0.85; 0.95]). We did not find any evidence to support the hypothesis of a lower adherence to generic bisphosphonates. In fact, prescribing of generic bisphosphonates led to a higher persistence rate and to better implementation at 1 year.

## Introduction

Osteoporosis is characterized by reduced bone mass and disruption of bone architecture, resulting in increased bone fragility and increased fracture risk^1^. Osteoporosis has been identified as a major public health concern due to the serious consequences of osteoporotic fractures and its increasing prevalence with population ageing^2–4^. The main objective of anti-osteoporotic treatment is to reduce the risk of fracture^5,6^. Bisphosphonates are antiresorptive medications which have demonstrated their efficacy to prevent osteoporotic fracture^7–10^. Oral bisphosphonates are recommended among the first-line treatments for osteoporosis^5,6^. Several studies have indicated that non-persistence and poor implementation were associated with increased risk of fracture^11,12^ and reduced drug effectiveness, which consequently results in significant higher clinical burden and healthcare costs^13^. As the problem of low adherence to osteoporosis treatment has been recognized worldwide, it is necessary to gather information on factors potentially associated with low implementation or persistence.

Adhering to a treatment regimen has been recently conceptualised as a process that includes three components. The first is starting the treatment -“initiation”-, the second is continuing to administer the treatment -“persistence”-, and the third is taking the treatment according to the recommended dosing regimen -“implementation”^14^. This taxonomy replaces previous terminology (e.g. compliance), which has been considered insufficiently precise for describing the temporal dynamics of adherence and its different causal influences and effects.

While medication costs are increasing, the use of less expensive generic versions of brand-name medications is considered by many National Healthcare Insurance Systems as an opportunity to contain costs. In France, a generic substitution policy that requires lower-cost generic drugs to be dispensed when they are available was set up in 2012^15^. The marketing authorization of a generic drug is based on the proof of bioequivalence but excipients may differ^16^. These changes in bisphosphonates formulations could lead to differences in pharmacokinetic properties between brandand generic drug. Indeed, several *in vitro* studies have shown that generics had greater bio-adhesive properties and had a different disintegration time^17,18^. These *in vitro* results could have direct tolerance consequences in increasing the upper gastrointestinal tract irritation and ulceration risk when generics are taken^19^.

Therefore the tolerability of generic may be worse than that of brand bisphosphonates, which could decrease medication adherence. Several studies found declines in treatment adherence with generic forms of bisphosphonates compared to branded forms^20–22^, while a study did not support this relation^23^ and another did not find differences in adverse events between the two forms^24^.

As this topic remains controversial we conducted an analysis of the French national database to examine whether the initiation of generic bisphosphonates resulted in lower adherence (implementation and persistence) compared to brand bisphosphonates.

## Results

### Patient characteristics

**Figure 1:**
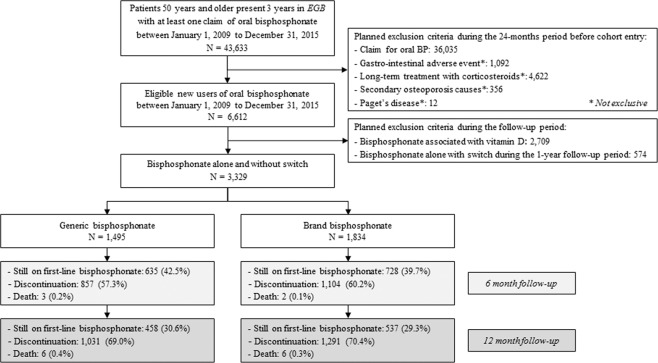
Flow chart of the study. *EGB* = Generalist Sample of Beneficiaries *(“Echantillon Généraliste des Bénéficiaires”)*.

Among 1,338,973 patients present in the Generalist Sample of Beneficiaries *(“Echantillon Généraliste des Bénéficiaires” or EGB)* during the 2009–2015 period, 43,633 aged 50 years or more had a reimbursement for oral bisphosphonate. Among them, 6,612 patients initiated an oral bisphosphonate treatment for primary osteoporosis, 2,193 (33.2%) with brand bisphosphonate, 1,710 (25.9%) with generic bisphosphonate, and 2,709 (41.0%) with bisphosphonate associated with vitamin D (Fig. 1).

Initiation of oral bisphosphonate treatments decreased over the 7-year study period (1,254 in 2009; 1,095 in 2010; 1,111 in 2011; 880 in 2012; 767 in 2013; 788 in 2014; and 717 in 2015). This was associated with a dramatic decrease of the proportion of “brand bisphosphonates” initiated from 58.1% in 2009 (728/1,254) to 8.2% (59/717) in 2015, and a concomitant increase in the proportion of “generic bisphosphonates” from 8.8% (110/1,254) to 49.1% (352/717).

**Figure 2:**
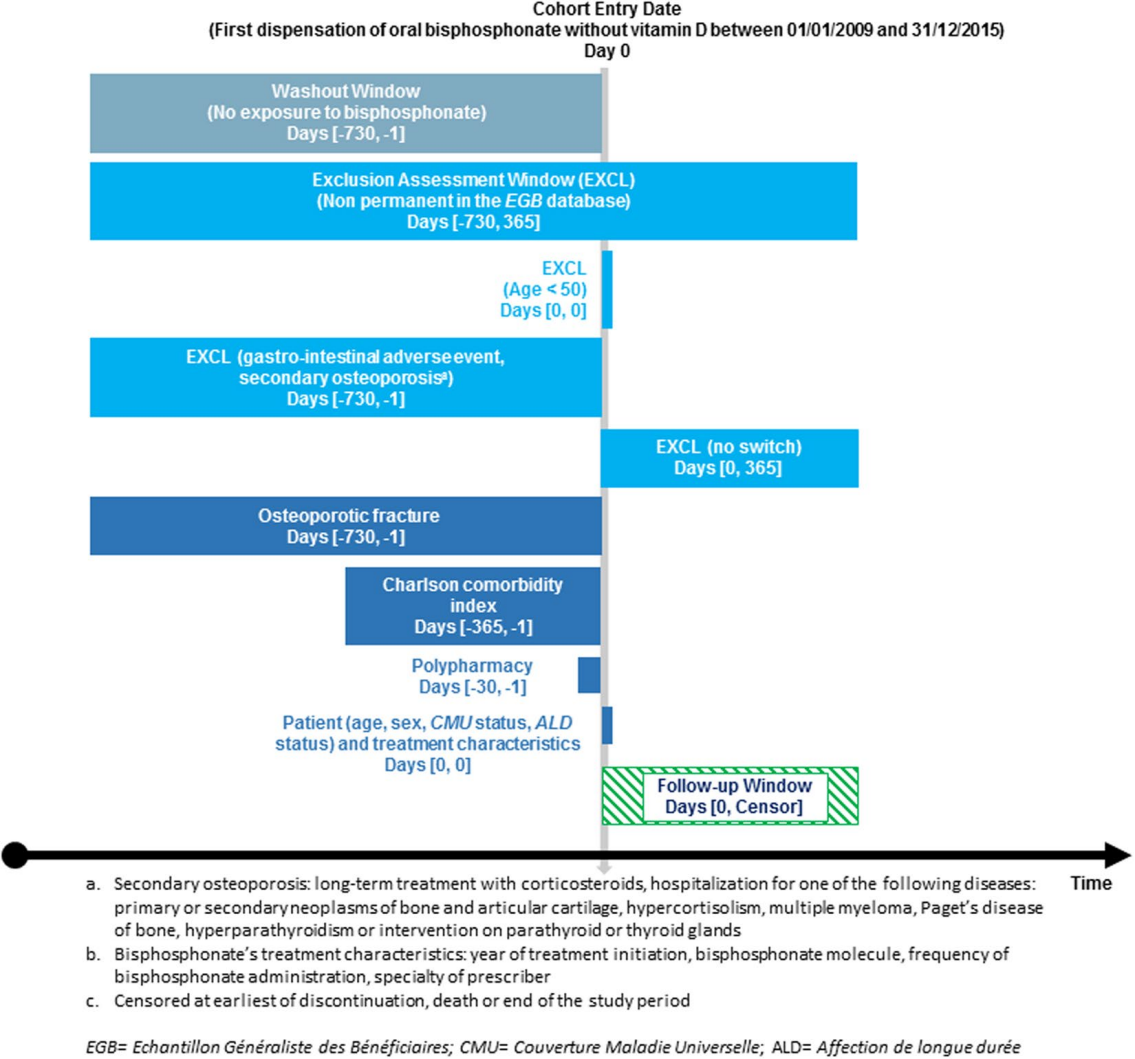
Study design.

A total of 232 (10.5%) patients who initiated with brand bisphosphonate and 156 (9.1%) who initiated with generic bisphosphonate switched from brand to generic (or conversely) of the same drug during the 12 months after initiation; while 127 (5.8%) who initiated with brand bisphosphonate and 59 (3.5%) who initiated with generic bisphosphonate changed to another bisphosphonate. After exclusion of patients who switched, the study population was composed of 1,834 patients in the “brand bisphosphonate” group and 1,495 in the “generic bisphosphonate” group (Fig. 2).

Patient characteristics are described in Table 1 according to the bisphosphonate group. Mean age was respectively 69.9 and 71.1 years in brand and generic bisphosphonate groups (p = 0.002). There was 90.6% of women in the “brand bisphosphonate” group and 87.6% in the “generic bisphosphonate” group (p = 0.01). More patients had a history of osteoporotic fracture in the “generic bisphosphonate” group than in the “brand bisphosphonate” group (respectively 7.0% and 4.1%; p < 0.001).

**Table 1:** Baseline characteristics of patients and description of initiated oral bisphosphonate treatments according to bisphosphonate group (N = 3,329).

	Brand bisphosphonate (N = 1,834)	Generic bisphosphonate (N = 1,495)	p-value^a^
**Characteristics of patients**			
Mean age (years) ± SD	69.9 ± 10.4	71.1 ± 10.5	0.002
Class of age (years), n (%)			0.01
50 to 59	368 (20.1)	244 (16.3)	
60 to 69	536 (29.2)	443 (29.6)	
70 to 79	540 (29.4)	435 (29.1)	
≥80	390 (21.3)	373 (25.0)	
Sex(female), n (%)	1,661 (90.6)	1,310 (87.6)	0.01
*ALD* status^b^, n (%)	766 (41.8)	633 (42.3)	0.74
*CMU* status^c^, n (%)	39 (2.1)	44 (2.9)	0.13
Polypharmacy^d^, n (%)	1,299 (70.8)	1,042 (69.7)	0.48
History of osteoporotic fracture^e^, n (%)	75 (4.1)	105 (7.0)	<0.001
Charlson comorbidity index, n (%)			0.11
0	1,219 (66.5)	981 (65.6)	
1–2	609 (33.2)	507 (33.9)	
≥3	6 (0.3)	7 (0.5)	
**Description of initiated oral bisphosphonate treatments**			
Year of initiation, n (%)			<0.0001
2009	688 (37.5)	85 (5.7)	
2010	549 (29.9)	95 (6.4)	
2011	316 (17.2)	145 (9.7)	
2012	126 (6.9)	230 (15.4)	
2013	58 (3.2)	292 (19.5)	
2014	47 (2.6)	326 (21.8)	
2015	50 (2.7)	322 (21.5)	
Oral bisphosphonate molecule, n (%)			<0.0001
Alendronic acid	162 (8.8)	706 (47.2)	
Risedronic acid	982 (53.5)	781 (52.2)	
Ibandronic acid	677 (36.9)	0	
Etidronic acid	13 (0.7)	8 (0.5)	
Frequency of bisphosphonate administration, n (%)			<0.0001
Daily	40 (2.2)	26 (1.7)	
Weekly	735 (40.1)	1,207 (80.7)	
Monthly	1,059 (57.7)	262 (17.5)	
Physician specialty, n (%)			<0.0001
General practitioner	1,222 (66.6)	972 (65.0)	
Specialist practitioner	407 (22.2)	192 (12.8)	
Hospital practitioner	199 (10.9)	322 (21.5)	
Unknown	6 (0.3)	9 (0.6)	

^a^The comparisons were performed with a Wilcoxon test for age and χ² tests for all categorical variables.

^b^The *ALD* status identifies patients with a long-term disease coded according to the International Classification of Disease, 10th version classification system (ICD-10), as declared by their general practitioner and approved by a physician employed by the National Healthcare Insurance.

^c^The *CMU* status identifies patients with low income.

^d^The polypharmacy was defined as five or more medications (Anatomical Therapeutic Chemical codes) dispensed in the same month of the index date.

^e^History of osteoporotic fracture in the 24-months period before index date.

### Treatments initiated

Initiated oral bisphosphonates treatments are described in Table 1 according to bisphosphonate group. There was no dispensation of generic ibandronic acid because in France this molecule was available only over a short period of time. Administration frequency was different between the two groups (p < 0.0001), with more patients under weekly bisphosphonate in the “generic bisphosphonate” group than in the “brand bisphosphonate” group (respectively 80.7% and 40.1%). Regarding the prescriber’s specialty, the proportions were equivalent between the two groups for general practitioners but differences were observed for specialist and hospital practitioners (p < 0.0001). After using the inverse probability of treatment weighted (IPTW) method, characteristics of patients and treatments initiated were balanced between the matched groups, as the standardized differences can be considered as negligible (Evolution pre- and post-weighting is presented in the Appendix 1).

### Treatment persistence

Among the 1,834 patients in the “brand bisphosphonate” group, 537 (29.3%) remained on first-line bisphosphonate after 12 months of follow-up, whereas 1,291 (70.4%) discontinued treatment, and 6 (0.3%) died. Among the 1,495 patients in the “generic bisphosphonate” group, only 458 (30.6%) remained on first-line bisphosphonate after 12 months of follow-up, 1,031 (69.0%) discontinued treatment, and 6 (0.4%) died (Fig. 2). Median time to discontinuation was 103 days in “brand bisphosphonate” group and 119 days in “generic bisphosphonate” group. Six and 12-months persistence were respectively 39.7% (728/1834) and 29.3% (537/1834) in “brand bisphosphonate” group, and 42.5% (635/1495) and 30.6% (458/1495) in “generic bisphosphonate” group. Initiating oral bisphosphonate treatment with brand resulted in a higher risk of discontinuation within 12 months (Hazard Ratio - HR = 1.08; 95%CI = [1.02;1.14]) (Table 2).

**Table 2:** Association between first-line brand vs. generic bisphosphonates and adherence to treatment during the first year after bisphosphonates initiation (N = 3,329).

	Brand bisphosphonate N = 1,834	Generic bisphosphonate N = 1,495	Relative Risk [CI 95%]
Time to discontinuation (d), median [Q1; Q3]	103 [63;365]	119 [61;365]	1.08 [1.02; 1.14]^a,b^
Class of CMA7, n (%)^c^			
<90%	173/728 (23.8)	139/635 (21.9)	0.90 [0.85; 0.95]^a,d^
≥90%	555/728 (76.2)	496/635 (78.1)	

^a^Models excluded 15 patients due to missing prescriber specialty.

^b^Hazard-Ratio [CI 95%] for discontinuationestimated from weighted Fine and Gray’s model accounting for competing risk of death with IPTW weights assigned to each patient (reference = “generic bisphosphonate” group).

^c^This analysis included the 1,364 patients under bisphosphonate treatment during at least 6 months (728 patients in the “brand bisphosphonate” group and 635 in the “generic bisphosphonate” group).

^d^Risk ratio [CI 95%] for a good implementation estimated using weighted log–binomial regression model with IPTW weights assigned to each patient (reference = “generic bisphosphonate” group).

### Treatment implementation

Among the 1,364 patients under first-line bisphosphonate treatment lasting for at least 6 months, the mean duration of treatment was 334 days (±57) in the “brand bisphosphonate” group and 331 days (±59) in the “generic bisphosphonate” group. Mean continuous multiple-interval measure of medication availability version 7 (CMA7) was 0.931 (Standard Deviation - SD = 0.072) in “brand bisphosphonate” group and 0.939 (SD = 0.073) in “generic bisphosphonate” group.The rate of good implementation (CMA7 ≥ 0.90) was 76.2% in “brand bisphosphonate” group and 78.1% in “generic bisphosphonate” group, and the risk of good implementation was significantly lower in “brand bisphosphonate” group (Risk Ratio - RR = 0.90; 95% Confidence Interval - CI = [0.85; 0.95]) (Table 2).

## Discussion

In this large population-based study, we compared implementation and persistence with oral bisphosphonate treatment among newly-treated osteoporotic patients with either brand or generic bisphosphonate. We did not find any evidence to support the hypothesis of a lower adherence to generic bisphosphonates as we could expect from the literature. Conversely, prescribing of generic bisphosphonates led to a higher persistence rate and to better implementation at 1 year. We have to further explore the possible mechanisms explaining these effects, as it is important to understand the factors underlying patients’ or prescribers’ decisions to implement suboptimally bisphosphonate treatment in order to develop interventions to improve persistence.

In the retrospective patient chart analysis of Ringe JD *et al*.^20^, which included 186 women with postmenopausal osteoporosis who had started with once-weekly bisphosphonate, 1-year persistence was significantly lower with generic alendronate (68%) as compared to branded alendronate (84%) and branded risedronate (94%). However this study was conducted retrospectively on clinical charts of one hospital, which leads to a small sample size and a high risk of selection biases. In 2009, Sheehy O *et al*.^22^ showed in a cohort of 32,804 patients that the risk of discontinuation doubled in patients initiated with generic alendronate compared to those started on branded alendronate. In 2012, Ström O *et al*.^21^ investigated the association between automatic generic substitution and medication persistence in an open historical cohort of 36,433 patients who started treatment with alendronate. Between 2006 and 2009, the proportion of generic prescriptions increased from10.8% to 45.2%, and the proportion of patients persisting with alendronate treatment for 12 months fell from 66.9% to 51.7%, while no difference was observed in persistence with proprietary risedronate during the same period. Regarding the association between 1-year persistence and initiation of brand versus generic drug of oral bisphosphonate, our results differ from these three previous studies. Potential explanations may be related to differences in patient’s selection, study period, oral bisphosphonate molecules studied, designs of the studies, or use of propensity scores.

Firstly, it is probable that over time and institutional campaigns, generic treatments have been progressively better accepted by the patients and the physicians who actually highly increased generic prescriptions over the years. As most previous studies were conducted before 2012 this might explain apparent discrepancies. Furthermore, our results showed an important association between year of treatment initiation and 1-year persistence, so we integrated this factor in propensity scores. Analyses have not been adjusted for this important confounding factor in all other studies and this could also partly explain the difference of results.

Secondly, we cannot exclude a potential confounding effect of the socio-economic status. From 2009 in France, the rules regarding prescription of generic drug were more restrictive and patients who were asking for a brand medication instead of generic had to advance the costs of the treatment while the generic medication was paid directly by the health insurance. This may partly explain a decrease of adherence in some patients who wanted to stay on brand medication but could not afford it.

Thirdly, associations could have been confounded by indication as it is possible that physicians, under the pressure of prescribing more generics, may have mostly prescribed generic to patients they considered likely to be more adherent, and brand bisphosphonate to patients likely to be less adherent.

Our results show a high implementation rate, over 90%, to newly-initiated oral bisphosphonates, among patients who were still on first-line bisphosphonate treatment during at least 6 months. This rate is not consistent with those presented in the meta-analysis of Imaz *et al*.^11^ which presented a mean medication possession ratio of 67%. This difference could be due to the fact that medication possession ratio evaluates both persistence and implementation in the same index, while we chose to evaluate the two dimensions separately. Direct comparison of implementation rates among published studies is difficult because of differences in study methodologies, enrollment criteria, adherence definitions, and follow-up period^25^. These difficulties highlight the necessity to precisely describe methodological choices for adherence evaluation, as the European Society for Patient Adherence, Compliance, and Persistence (ESPACOMP) Medication Adherence Reporting Guidelines (EMERGE) stated^26^.

The main strength of this study is that it was based on a recent population-level representative sample of the exhaustive national reimbursement database^27^, over a seven-year period. The validity and usefulness of this database in pharmaco-epidemiologic studies have been previously studied and validated^28,29^. The analysis of large medico-administrative databases offers advantages of completeness of patients and unbiased study sample.The second strength of this study is the evaluation of implementation using CMA7 which accounts for the timing of the dispensing events^30^, and our calculation which adjusted for hospitalization.

Our study has several limitations due to the nature of the available data^31^. Firstly, our database contained no information on drug indication. Therefore, we had to use proxies to exclude patients who might have used secondary osteoporosis treatment. Secondly, assessment of medication adherence based on records of dispensed drug supplies could lead to its overestimation. However, Noize *et al*.^32^ have found that agreement between reimbursement data contained in the French Healthcare Insurance System database and the self-reported drug use at interview of patients was good for drug treatments for bone disease (Kappa = 0.73; 95%CI = [0.67–0.79]). Thirdly, the use of the defined daily doses as a proxy for the prescribed daily doses of bisphosphonates could have led to outcome misclassification^33^. Finally, no information about the reason for discontinuing treatment was available. Therefore, the study population might contain non-persistent patients who discontinued drug therapy due to their physician’s advice.

## Methods

A new-user cohort study was carried out using the nationwide representative sample of the French National Healthcare Insurance database (*EGB*) (Fig. 2). We used the ESPACOMP medication adherence reporting guidelines (EMERGE)^26^ and the reporting of studies conducted using observational routinely collected health data statement for pharmacoepidemiology (RECORD-PE) statement^34^ to guide the reporting.

### Data source

The *EGB* is a 1/97th random permanent representative sample of all beneficiaries covered by the National Health Insurance, with planned 20-year longitudinal data^35^. The *EGB* included in 2017 about 780,000 people. It contains anonymous demographic data and reimbursement data for physician or paramedical visits, medicines, medical devices, and lab tests (without results); chronic medical conditions (International Classification of Disease, 10th version -ICD-10- codes); hospitalisations with ICD-10 codes for primary, linked and associated diagnoses, date and duration, procedures, diagnostic‐related groups, cost, and date of death.

Since the study was strictly observational and used anonymous data, in accordance to the laws that regulate “non-interventional clinical research” in France, the written informed consent from the participants or the authorization from any other ethics committee were not required to conduct this study.

### Study population

We included all patients aged 50 and older, present at least 3 years in the *EGB* database, who were new users of oral bisphosphonates for primary osteoporosis between 01/01/2009 and 31/12/2015. The index date was defined by initiation of oral bisphosphonate use (dispensation of alendronic acid, ibandronic acid, risedronic acid or etidronic acid, without association with vitamin D) after at least 24 months without any dispensing of study drugs. Patients initiating oral bisphosphonate treatment with bisphosphonate associated with vitamin D were not included because no generic drug was commercialized during the study period for these combinations. Patients who switched (from generic to brand or from brand to generic oral bisphosphonate, or from one oral bisphosphonate to another) were not included. The first dispensation of oral bisphosphonate defined the group to which the patients were assigned. The group “generic bisphosphonate” included all patients with a generic oral bisphosphonate initiation, the group “brand bisphosphonate” included all patients with a brand oral bisphosphonate initiation. Patients were followed from the initiation date (index date) up to 12 months.

To identify patients with primary osteoporosis, patients were not included if they were receiving long-term treatment with corticosteroids (3 reimbursements or more) or if they were hospitalized for one of the following diseases over the 24-month period before the index date: primary or secondary neoplasms of bone and articular cartilage (ICD-10 diagnosis code C79.5), hypercortisolism (E24), multiple myeloma (C90.0), Paget’s disease of bone (M88), gastro-intestinal adverse event (K20.x–K21.x, K22.1–K22.3, K22.6, K22.8, K25.x–K28.x, K29.0, K29.7–K29.9, K30.x, K92.0–K92.2); hyperparathyroidism or intervention on parathyroid or thyroid glands (E05, E06, E21, excluding E21.4 and E21.5, or medical or surgical procedure codes).

We extracted the following demographic and clinical patients’ characteristics from the database: date of birth, *CMU* (*Couverture Maladie Universelle*) status which identifies patients with low income, and *ALD* (*Affection de longue durée*) status. The *ALD* status identifies patients with a long-term disease (LTD) coded according to the ICD-10, reported by their general practitioner and approved by a physician of the National Healthcare Insurance. Comorbidity was estimated by the Charlson comorbidity index, a commonly-used proxy previously validated in the National French Health Insurance System^36,37^. The Charlson comorbidity index was calculated over the past 12 months before inclusion through ICD-10 codes of in-hospital or LTD diagnoses, and reimbursement of specific medications. Polypharmacy was defined as five or more medications (Anatomical Therapeutic Chemical codes) dispensed in the same month of the index date. History of osteoporotic fracture was considered for patients with a hospitalization for hip (S72.0, S72.00, S72.1, S72.10, S72.2, S72.20)^38^, vertebral (S22.0, S22.00, S22.1, S22.10, S32.0, S32.00, S32.7, S32.70), humerus (S42.2, S42.20), forearm (S52.5, S52.50, S52.6, S52.60), tibia (S82.1, S82.10) or fibula (S82.4, S82.40) fracture in the 24-month period before the index date.

To describe the initiated oral bisphosphonates treatments, we extracted the following data from the database: year of initiation (2009 to 2015), oral bisphosphonate molecule (alendronic, ibandronic, risedronic or etidronic acid), frequency of bisphosphonate administration (daily, weekly or monthly), and specialty of the first prescriber of bisphosphonate (general, specialist or hospital practitioner).

### Study measures

Adherence to oral bisphosphonate was evaluated through two components: whether the patients took their medication in accordance to the recommended dosing regimen (implementation) and whether they continued taking it for the recommended duration (persistence).

Persistence was measured as the time to discontinuation and as the proportion of patients still on treatment at 6 and 12 months. Patients were allowed to have gaps between filled prescriptions, and were defined as non-persistent if they had no new dispensation during a period greater than 2 times the duration of the previous supply. The approach accounted for a variability of supply duration dependant on the size of the drug package, which may cover either 1 or 3 months of treatment. If a gap between refills included a hospitalization, we deducted the number of days of hospital stay from the gap duration. In case of non-persistence, the time to discontinuation was evaluated as the number of days between initiation date and the date of the last dispensing plus the number of days’ supply. Patients who died were censored at their death date.

Implementation was evaluated using the continuous multiple-interval measure of medication availability (CMA measure) version 7^30^, for patients under treatment during at least 6 months (time from initiation to discontinuation). As measure of CMA could be highly overestimated for treatment lasting less than 6 months, we did not include these patients in implementation analysis. This index takes into account the timing of dispensing events and identifies the number of days without medication supply (gap days) between these events assuming 100% adherence until supply ends. It also carries over any extra supply at the date of a new event to the next, which takes into account early refills. Dosages considered for the CMA calculation were the defined daily doses^39^, which correspond to the most common prescription for bisphosphonate. Implementation was calculated over the period of persistence, between the initiation date (index date) and either the date of bisphosphonate discontinuation (calculated by adding to the date of the last dispensation, the number of days covered by it), the date of death, or 12 months. When patients were hospitalized, we assumed that treatments were supplied by the hospital during the entire hospitalization period: we added all hospitalized days to the number of days covered, and remaining supplies were extended accordingly. A good implementation was defined as CMA7 ≥ 0.9.

### Statistical analyses

Demographic and clinical characteristics of incident oral bisphosphonates users and characteristics of treatments initiated were summarized according to bisphosphonate groups. Continuous variables were described using means and standard deviations and categorical variables with numbers and percentages. Comparisons were performed between “brand bisphosphonate” and “generic bisphosphonate” groups with Wilcoxon tests for continuous variables and χ² tests for categorical variables.

A propensity score-based analysis was performed using the inverse probability of treatment weighted (IPTW) method^40^ to balance the distribution of measured potentially confounding factors between the “brand bisphosphonate” and the “generic bisphosphonate” groups. Weights were derived from propensity score estimated as the predicted probability of a patient to initiate treatment with brand bisphosphonate using a logistic regression model, including the following covariates: sex, age (continuous), *CMU* status (yes or no), polypharmacy (yes or no), Charlson comorbidity index (0, 1–2, ≥3), history of osteoporotic fracture (yes or no), year of initiation (2009 to 2015), frequency of bisphosphonate administration (daily, weekly or monthly), and specialty of the first prescriber of bisphosphonate (general, specialist or hospital practitioner) as well as an interaction term between year of initiation and frequency of bisphosphonate administration.

The IPTW was stabilised with the marginal prevalence of receiving the treatment^41^. Standardized differences were used to assess the degree of balance between the matched groups for baseline characteristics^42^. An absolute standardized difference ≤ 0.10 was chosen to indicate a negligible difference in the mean or prevalence of a variable between groups. Balance for continuous variables was also assessed using graphical methods (side-by-side boxplots, empirical cumulative distribution functions, empirical QQ-plots) to compare the distributions across the 2 groups.

To compare risk of discontinuation (non-persistence) between the “brand bisphosphonate” and the “generic bisphosphonate” groups, hazard ratios (HR) was estimated between initiation and 12 months after, from weighted Fine and Gray’s model accounting for competing risk of death with IPTW weights assigned to each patient. To compare implementation between the two groups, risk ratio (RR) for a good implementation was estimated using weighted log–binomial regression model with IPTW weights assigned to each patient. Point estimates were provided along with 95% confidence interval (CI). All analyses were two-sided, and a p-value of less than 0.05 was considered statistically significant. Data manipulation and analyses were performed with Statistical Enterprise Guide software (SAS Institute, Cary, NC).

## Supplementary information

Appendix 1.

## Data Availability

The data that support the findings are available from CNAMTS, but restrictions apply to the availability of these data, which were used under license for the current study, and so are not publicly available.
