# Supplementary material for: Adherence with brand versus generic bisphosphonates among osteoporosis patients: a new-user cohort study in the French National Healthcare Insurance database
Source: Sci Rep. 2020 May 4;10:7446. doi: 10.1038/s41598-020-64214-x (PMC7198539; doi:10.1038/s41598-020-64214-x)
Supplement: Supplementary file 1 — Appendix 1. [file 41598_2020_64214_MOESM1_ESM.pdf]

Title: Adherence with brand versus generic bisphosphonates among osteoporosis patients: a new-user cohort study in the French National Healthcare Insurance database

Authors names and affiliations: Viprey Marie[1,2], Xue Yufeng[2], Rousseau Aurélie[3], Payet Cécile[1,2], Chapurlat Roland[4,5], Caillet Pascal[6], Dima Alexandra[2], Schott Anne-Marie[1,2]

[1] Hospices Civils de Lyon, Pôle de Sante Publique, Lyon, France

[2] Univ. Lyon, Université Claude Bernard Lyon 1, HESPER EA 7425, Lyon, France

[3] Centre Hospitalier de Bourg en Bresse, Service pharmaceutique, Bourg en Bresse, France

[4] Université de Lyon, INSERM UMR 1033, Lyon, France

[5] Hospices Civils de Lyon, Hôpital Edouard Herriot, Service de Rhumatologie, Lyon, France

[6] CHU de Nantes, Service de Pharmacologie Clinique, Nantes, France

Corresponding author:

Pr Anne-Marie Schott, MD, PhD

Pôle de Santé Publique, Hospices Civils de Lyon, 162 avenue Lacassagne, F-69003, Lyon, France; Univ. Lyon, Université Claude Bernard Lyon 1, HESPER EA 7425, 8 avenue Rockefeller, F-69008 Lyon, France; +33 (0) 4 72 11 57 70; anne-marie.schott-pethelaz@chu-lyon.fr

Appendix 1: Characteristics of patients and treatment initiated pre- and post-weighting

	Standardized difference pre-weighting	Standardized difference post-weighting
Age (years)	-0.11	<0.01
Sex	0.09	-0.04
CMU status[1]	-0.05	0.04
Polypharmacy[2]	0.09	0.06
History of osteoporotic fracture[3]	-0.13	0.03
Charlson comorbidity index	0.15	0.09
Year of initiation	1.75	0.04
Frequency of bisphosphonate administration	0.93	0.02
Physician specialty	0.35	0.09

[1] The *CMU* status identifies patients with low income
[2] The polypharmacy was defined as five or more medications (Anatomical Therapeutic Chemical codes) dispensed in the same month of the index date
[3] History of osteoporotic fracture in the 24-months period before index date
